# Radiation exposure to staff members during traction X-rays

**DOI:** 10.1186/1748-7161-8-S1-P16

**Published:** 2013-06-03

**Authors:** P Knott, M McGirk, A Markovic

**Affiliations:** 1 Rosalind Franklin University, Chicago, USA

## Background

Determining the flexibility of a scoliosis curve is important, during both conservative and surgical management, and traction x-rays of the patient’s spine are frequently done as part of this evaluation. The traction x-ray is done with the patient in the supine position, and with staff members pulling on the patient’s arms and legs. The use of staff to produce the traction radiograph has two major disadvantages: the pull on the arms and legs is not standardized, and the staff is exposed to x-ray each time they perform a radiograph. Ingegno [1] determined that exposure to personnel applying arm traction, for cervical radiographs, was 0.01mGy. But there are few studies that assess the radiation exposure to staff during imaging for AIS. It has been determined that a typical spinal radiograph exposes the patient to 3.2 (+/-1.6) mGy of radiation. [2]

## Aim

The aim of this study was to measure the amount of radiation a staff member is exposed to while participating in a traction x-ray of a patient with AIS.

## Methods

The x-ray table was set up and a phantom block of plastic was used to provide the same x-ray scatter as a human body. An electronic x-ray survey detector was used at the head and foot of the table, in the same position that a staff member’s head would be during traction. Radiographs were taken, and measurements of the amount of scatter were recorded.

## Results

The scatter produced was 0.003 to 0.009 mGy at the level of the staff member’s head. Given that staff wears a lead apron, the torso dose would be negligible, so the exposure to the eyes and thyroid would be the most clinically important.

## Conclusion

The recommended limit of exposure to the eyes for a staff member is 150 mGy per year [2]. With the exposure that we measured, the staff member would not reach this limit until they performed 17,000 to 50,000 procedures. However, since this dose of x-ray to the staff member has no therapeutic benefits, it should still be reduced whenever possible.
